# Editorial: The State of Science and Innovation of Bioactive Research and Applications, Health, and Diseases

**DOI:** 10.3389/fnut.2019.00178

**Published:** 2019-11-25

**Authors:** Alessandra Durazzo, Massimo Lucarini

**Affiliations:** CREA-Research Centre for Food and Nutrition, Rome, Italy

**Keywords:** bioactive molecules, nutrients, nutraceuticals, bioactive components, food chains, innovation, applications

## Introduction

The biological and physiological functions of each food matrix derive from the concerted action of nutrient components and biologically active compounds. Beyond basic nutrition, the dietary components possess beneficial roles, that leads to the advancement of perception of food as functional and nutraceutical. In this Research Topic, new features of nutrients were considered and the boundaries between nutrients and bioactive compounds were explored.

The monitoring of the distribution of compound classes in food groups and at different stages of the food chain was considered. Particular attention was given to the categorization of the effects of different processes, storage methods, technologies, packaging systems, etc.; in this regard conventional and innovative methods as well as multi-aspects and factors were taken into consideration.

As instance, Khoo et al. described characterized *Canarium odontophyllum* fat extracted using supercritical carbon dioxide, with focus on fatty acid profile.

Evidence of the benefits/risks of nutrients and bioactive compounds were included, taking into account the challenge of, and need for, new biomarkers and innovative assessment methodologies. The new role of nutrition based on a multi-factors approach were also considered.

In this regards it is worth mentioning the review of Norwitz et al. on the D-β-Hydroxybutyrate and its mechanism of action in the multiple cellular pathologies of Parkinson's disease. Another example is given by Kühn et al. on modulation on desaturase expression and fatty acid composition of cultured hepatocytes by resveratrol. It is well-known that there is an increase of number of factors, affecting the content in bioactive components, in line with the new characteristics of food chain.

At the same time, the understanding of activities and benefits of nutrients and bioactive compounds in humans is essential. Another focal point in this Research Topic was the uses and applications of bioactive components in the great range of fields, with particular regards nutraceuticals ones (1–3). Several papers, within this Research Topic, discuss the impact on new formulations and applications.

As instance, Sekhon-Loodu and Rupasinghe have given a snapshot of beneficial properties of some traditional medicinal plants, with focus on antioxidant, anti-diabetic, and anti-obesity potential. Also Boudreau et al. identified compounds with novel adipogenic bioactivity in distinct fractions of an *Artemisia scoparia* extract. On the other hand, Nguyen et al. characterized a probiotic beverage from pineapple juice fermented with *Lactobacillus* and *Bifidobacterium* strains.

Moreover, innovative applications of bioactive compounds in a technological scenario and nutritional needs, i.e., omics technologies, nanotechnologies, imaging techniques, chemometrics, mobile-based technologies were discussed. As instance, Kumar gives an overview of current status of peptides and peptidomimetics as potential anti-obesity agents.

## Author Contributions

All authors listed have made a substantial, direct and intellectual contribution to the work, and approved it for publication.

### Conflict of Interest

The authors declare that the research was conducted in the absence of any commercial or financial relationships that could be construed as a potential conflict of interest.
